# Exosome Biogenesis: Meta-Analysis of Intraluminal Vesicle Size Across Species

**DOI:** 10.3390/ijms27073176

**Published:** 2026-03-31

**Authors:** Sayam Ghosal, Rita Leporati, Bora Yilmaz, Brachyahu M. Kestecher, Bernadett R. Bodnár, Mohamed A. Fattah, Luigi Menna, Angéla Takács, Hargita Hegyesi, László Kőhidai, Edit I. Buzas, Xabier Osteikoetxea

**Affiliations:** 1 HCEMM-SU Extracellular Vesicle Research Group, 1089 Budapest, Hungary; 2 Institute of Genetics, Cell- and Immunobiology, Semmelweis University, 1089 Budapest, Hungary; 3 Division of Medical Oncology, Modena University Hospital, 41124 Modena, Italy; 4 HUN-REN-SU Translational Extracellular Vesicle Research Group, 1089 Budapest, Hungary

**Keywords:** ILV, MVB, sEV, exosome biogenesis, ESCRT, TEM, cryo-EM, dSTORM

## Abstract

Exosomes, a major subpopulation of small extracellular vesicles (sEV), are conserved mediators of intercellular communication, yet the properties of their endosomal precursors, intraluminal vesicles (ILV), have not been systematically quantified across species or imaging modalities. This study systematically evaluates ILV sizes across diverse eukaryotic species and modalities while assessing their relationship to secreted sEV sizes. We carried out two complementary meta-analyses of ILV sizes based on transmission electron microscopy (TEM) and cryogenic electron microscopy (cryo-EM) data across species. This was followed by in situ assessment of sEVs secreted by HEK293T cells with TEM, nanoparticle tracking analysis and super-resolution microscopy characterization. Across species, imaging modalities, and cellular contexts, ILV sizes were under approximately 200 nm, with a mean diameter of 100.5 nm, overlapping with the size range of sEVs. This study addresses an existing knowledge gap by systematically evaluating ILV size across species and revealing an upper size limit of approximately 200 nm.

## 1. Introduction

The field of extracellular vesicle (EV) research has progressed significantly in recent decades, elucidating the complex nature and increasing potential of EVs for disease diagnosis and therapy [1,2,3,4,5,6,7]. EVs have been shown to participate in both the progression and inhibition of various conditions, including cancer [8], cardiovascular disorders [4,9], and neurological diseases [10].

EV biogenesis is generally described as occurring through two principal pathways. In one route, vesicles, often termed ectosomes, are generated by direct outward budding from the plasma membrane. In the second route, vesicles termed exosomes are formed as intraluminal vesicles (ILVs) within multivesicular bodies (MVBs) or amphisomes, which are subsequently released into the extracellular space following fusion of the limiting membrane of MVBs or amphisomes with the plasma membrane [11,12,13,14]. ILV formation of MVBs is mediated by the endosomal sorting complexes required for transport (ESCRT), which coordinate cargo recognition and membrane scission, while lipid-rich microdomains and membrane curvature drive the inward invagination. ESCRT independent mechanisms involving lipid microdomains, ceramide, and tetraspanins generate structurally distinct ILV populations with potentially different sizes, indicating multiple molecular routes to ILV biogenesis [15,16,17].

EV secretion has been reported across all domains of life [18]. Yet, the quantitative properties of ILVs, particularly their size, have not been systematically evaluated across species. Although the molecular mechanisms of ILV biogenesis have been extensively characterized, it remains unclear whether ILVs generated through ESCRT-dependent or ESCRT-independent pathways exhibit common sizes across organisms. Reported ILV sizes vary considerably in the literature, most commonly ranging from 30 to 150 nm [19,20] depending on cell type and detection methodology, although larger vesicles have occasionally been reported, and the impact of imaging modality has not been systematically compared. Transmission electron microscopy (TEM) is predominantly used, while cryogenic electron microscopy (cryo-EM), which avoids fixation artifacts and better preserves native membrane architecture, has been applied less frequently.

In this study, a comprehensive and integrative approach was undertaken to address these gaps. Two independent meta-analyses were performed to evaluate ILV sizes across species based on published TEM and cryo-EM-based studies, enabling direct comparison between imaging modalities. In addition, sEVs were isolated from HEK293T cells and characterized to examine the relationship between ILV dimensions and the size of secreted vesicles. Together, these analyses were designed to establish a quantitative framework for ILV size conservation and to clarify its relationship to exosome and sEV sizes across systems.

## 2. Results

### 2.1. Conserved Intraluminal Vesicle Size Across Species Revealed by TEM Meta-Analysis

**Figure 1:**
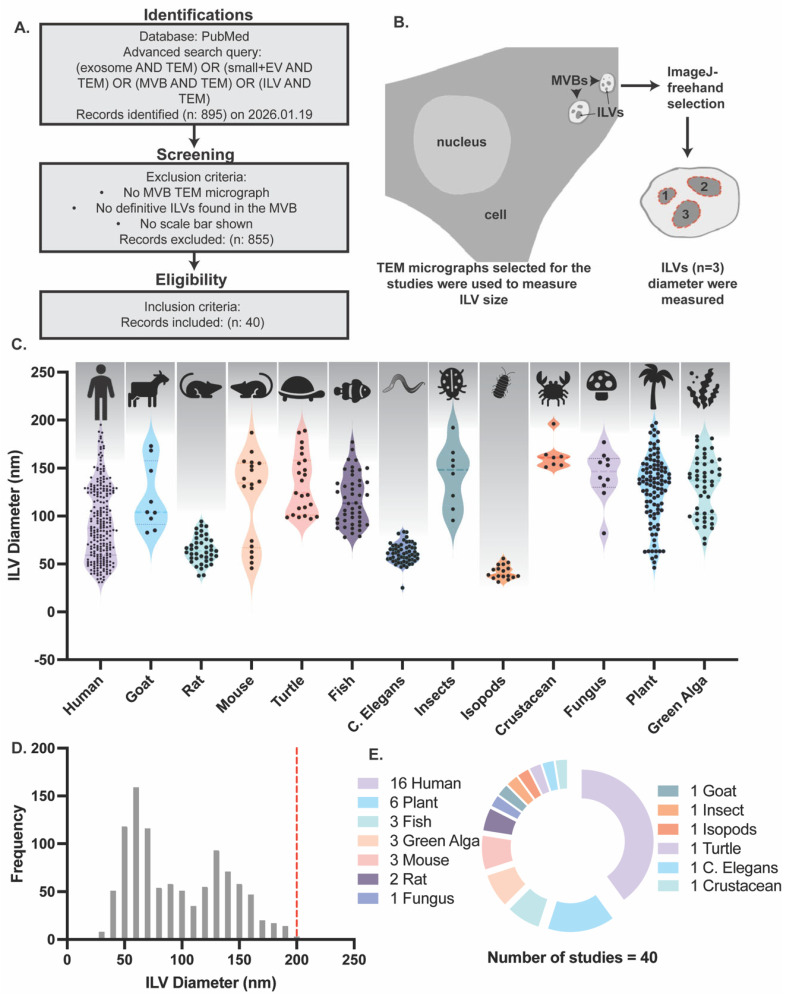
Meta-analysis of intraluminal vesicle (ILV) size measured by TEM across species. (**A**) PRISMA-based workflow illustrating the literature search, screening, and inclusion strategy used to identify TEM-based studies reporting ILV measurements. A total of 895 records were retrieved from PubMed, of which 40 studies met the inclusion criteria. (**B**) Schematic representation of ILVs was measured in ImageJ (v1.54g) in situ images after scale calibration, by freehand tool selection and Feret diameter quantification. (**C**) Violin plots show the distribution of ILV diameters across species derived from TEM micrographs included in the meta-analysis. Descriptive statistics are summarized with the symbols for the respective species as follows: human (*N* = 245; mean = 92.87; SD = 38.97), goat (*N* = 9; mean = 119.6; SD = 34.57), rat (*N* = 41; mean = 64.82; SD = 14.13), mouse (*N* = 18; mean = 119.4; SD = 45.84), turtle (*N* = 23; mean = 133.3; SD = 30.85), fish (*N* = 46; mean = 117.1; SD = 26.28), c. elegans (*N* = 59; mean = 61.05; SD = 10.34), insects (*N* = 8; mean = 141.9; SD = 32.28), isopods (*N* = 17; mean = 41.62; SD = 6.994), crustacean (*N* = 7; mean = 163; SD = 15.38), fungus (*N* = 10; mean = 142.4; SD = 26.49), plant (*N* = 102; mean = 128.4; SD = 35.54), green alga (*N* = 46; mean = 131.3; SD = 30.56). Here, N is the number of ILV measured. (**D**) Frequency distribution of ILV diameters measured from TEM micrographs across all included studies. The distribution exhibits a unimodal profile with a right-skewed tail. The dashed red line indicates the 200 nm upper size reference, above which only a small fraction of ILVs were observed. (**E**) A burst pie chart illustrating that most peer-reviewed articles studied human samples, followed by plant, mouse, fish, algae, and rat. The number represents the number of studies per species.

To determine whether ILV size varies across species, a comprehensive meta-analysis of previously published studies indexed in PubMed was performed. Two independent meta-analyses were conducted. In the first analysis, only studies in which ILVs were characterized by using TEM were used. For each selected study, ILV diameters were estimated from TEM micrographs using scale bar calibration (Supplementary Information Figure S1). Individual ILVs within MVBs were measured, and the organism used in each study was recorded. The number of ILVs measured (N), mean diameter, standard deviation (SD), and lower and upper limits of the 95% confidence interval (CI) were calculated and summarized in Table 1. ILVs were analyzed across a broad range of species, including human, goat, rat, mouse, turtle, fish, Caenorhabditis elegans, insects, isopods, crustaceans, fungi, plants, and green algae (Figure 1C). Across all species, the mean ± SD of ILV diameter was 112.05 ± 26.79 nm. The largest ILVs were observed in plant-derived samples, with diameters reaching up to 197 nm, whereas the smallest ILVs, measuring approximately 25 nm in diameter, were observed in Caenorhabditis elegans. Notably, the upper limit of ILV diameter remained below 200 nm across all species examined. The frequency distribution of ILV diameters exhibited a unimodal, right-skewed profile, with the majority of ILVs measuring between approximately 40 and 120 nm (Figure 1D). The distribution peaked around 60–80 nm, and progressively fewer ILVs exceeded 100 nm in diameter. The dashed red line at 200 nm indicates that all measured ILVs were smaller than this threshold. Human-derived studies constituted the largest proportion of the dataset, followed by studies in plants, fish, green algae, mice, and rats. Additional taxa—including fungi, goat, insects, isopods, turtle, Caenorhabditis elegans, and planktonic crustaceans—were each represented by a single study, highlighting the phylogenetic diversity of biological systems in which ILVs were analyzed (Figure 1E). Collectively, ILVs measured from TEM micrographs across all examined species were consistently observed to be smaller than 200 nm in diameter.

### 2.2. Conserved Intraluminal Vesicle Size Across Species Revealed by Cryo-EM Meta-Analysis

**Figure 2:**
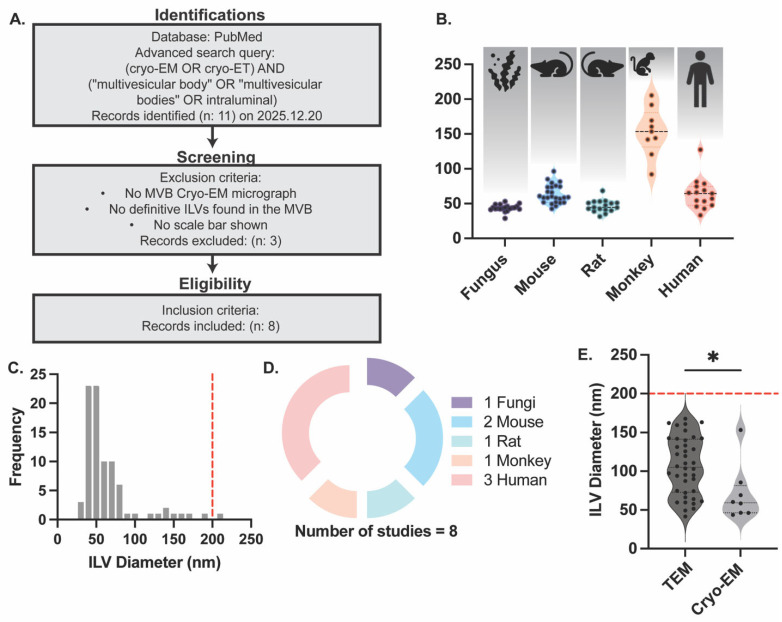
Meta-analysis of intraluminal vesicle (ILV) size measured by cryo-EM and comparison with TEM-derived measurements. (**A**) PRISMA-based workflow illustrating the literature search, screening, and inclusion strategy for cryo-EM-based studies reporting ILV measurements. A total of 11 records were retrieved from PubMed, of which 8 studies met the inclusion criteria. (**B**) Violin plots showing species-specific distributions of ILV diameters measured by cryo-EM across species as showed with symbol respectively. Descriptive statistics are summarized as follows: human (*N* = 15; mean = 64.05 nm; SD = 22.27 nm; 95% CI = 51.72–76.39 nm), monkey (*N* = 9; mean = 153.3 nm; SD = 34.58 nm; 95% CI = 126.7–179.9 nm), rat (*N* = 7; mean = 46.38 nm; SD = 8.675 nm; 95% CI = 41.92–50.85 nm), mouse (*N* = 5; mean = 63.50 nm; SD = 13.42 nm; 95% CI = 57.96–69.04 nm), and fungus (*N* = 2; mean = 43.68 nm; SD = 5.074 nm; 95% CI = 41.31–46.06 nm). Here, N is the number of ILV measured per species. (**C**) Frequency distribution of pooled ILV diameters measured by cryo-EM across all included studies. The distribution exhibits a unimodal profile centered in the sub-100 nm range. The dashed red line denotes the 200 nm upper size reference. (**D**) Burst pie chart illustrating the taxonomic composition of the cryo-EM dataset, indicating the relative contribution of human, monkey, mouse, rat, and fungal studies to the pooled analysis. (**E**) Comparison of ILV diameters measured by TEM and cryo-EM. Statistical analysis was performed using an unpaired Welch’s *t*-test (two-tailed). Mean ILV diameter measured by TEM was 109.6 nm, whereas the mean ILV diameter measured by cryo-EM was 70.37 nm. The difference between means was 39.25 ± 14.12 nm, with a 95% CI of 7.859–70.65 nm. The difference was statistically significant (* = *p* < 0.005). Sample sizes were *N* = 39 for TEM and *N* = 8 for cryo-EM. The dashed red horizontal line indicates the 200 nm upper size reference.

To assess whether variability in ILV size may arise from technical limitations associated with TEM, a second meta-analysis was performed focusing on ILV measurements obtained using cryo-EM. Individual ILVs within MVBs were measured, and the organism used in each study was recorded. The number of ILVs measured (N), mean diameter, SD, and lower and upper limits of the 95% CI were calculated and summarized in Table 2. ILVs were analyzed across multiple species, including human, monkey, rat, mouse, and fungi (Figure 2B). The majority of ILV diameters were below 200 nm; however, a single ILV measured in a monkey-derived sample exhibited a diameter of 205.54 nm, representing 1 out of 86 ILVs analyzed. The frequency distribution of ILV diameters derived from cryo-EM datasets exhibited a narrow, unimodal profile, with most ILVs measuring between approximately 40 and 90 nm (Figure 2C). The distribution peaked around 50–60 nm, and only a single ILV exceeded 200 nm, while most measurements fell within the 200 nm upper limit indicated by the dashed red line. The species distribution of studies included in the cryo-EM meta-analysis is shown in Figure 2D. Human-derived studies constituted the largest proportion of the dataset, followed by mouse-derived studies, whereas monkey, rat, and fungal samples were each represented by a single study. Mean ILV diameters ± SD obtained from TEM and cryo-EM-based studies were compared, revealing that ILVs measured by TEM were significantly larger and had higher variability than those measured by cryo-EM (Figure 2E). Comparison of the two imaging modalities’ statistics is shown in Table 3. The average mean ± SD of ILV diameter of combined studies is 100.5 ± 39.12 nm. Despite this difference, cryo-EM-derived ILV sizes remained predominantly below the 200 nm upper size limit and were largely consistent with TEM-based measurements.

### 2.3. Comparison of ILV and sEV Size Using Orthogonal Methods

**Figure 3:**
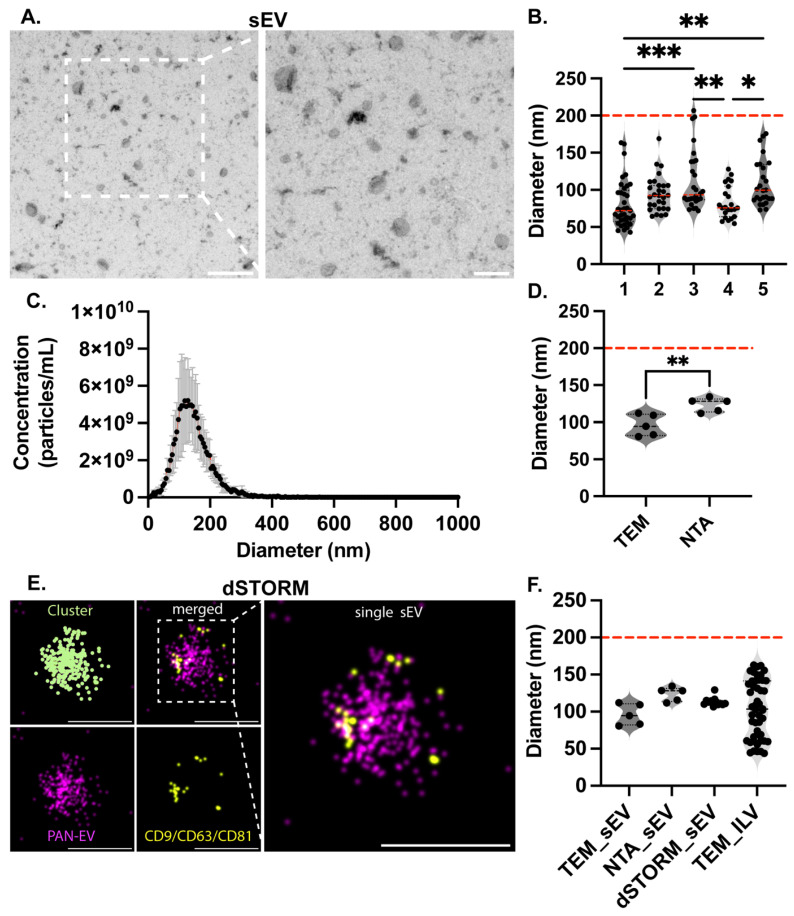
Characterization of sEV size and comparison with ILV dimensions. (**A**) Representative TEM micrographs of sEVs isolated from HEK293T cells. The left panel shows a low-magnification field (scale bar: 500 nm), and the right panel shows a magnified view highlighting the characteristic cup-shaped, bilayer morphology of sEVs (scale bar: 200 nm). (**B**) sEV diameters measured from TEM micrographs across five biological replicates. Individual data points represent single vesicle measurements. Mean ± SD values for each biological replicate were as follows: replicate 1, 80.81 ± 31.51 nm (*N* = 37); replicate 2, 94.54 ± 23.97 nm (*N* = 29); replicate 3, 112.1 ± 39.91 nm (*N* = 28); replicate 4, 83.13 ± 22.01 nm (*N* = 23); replicate 5, 109.4 ± 31.15 nm (*N* = 26). Analysis of sEV diameters measured by TEM across biological replicates showed that biological replicate 3 exhibited significantly larger sEV diameters compared with biological replicates 1 and 4, whereas biological replicate 5 was significantly larger than biological replicates 1 and 4. Statistical significance was determined using ordinary one-way ANOVA followed by Tukey’s multiple comparisons test (Red dashed line showing 200 nm). (**C**) NTA-derived size distribution of sEVs across five biological replicates, plotted as mean ± SD particle concentration as a function of diameter. The distribution exhibited a unimodal profile with the highest particle concentration within the sub-200 nm range (Red dashed line showing 200 nm). (**D**) Comparison of mean sEV diameters measured by TEM and NTA for the same five biological replicates. Mean ± SD sEV diameter measured by TEM was 96.00 ± 14.47 nm, whereas mean ± SD diameter measured by NTA was 123.7 ± 9.507 nm. An unpaired two-tailed *t*-test revealed a statistically significant difference between TEM and NTA measurements. (**E**) Representative dSTORM images of individual sEVs showing phenotypic characterization. Single sEVs were detected as discrete clusters, with phosphatidylserine labeling (PAN-EV) delineating the vesicle membrane and co-localization of a mix of canonical tetraspanins CD9, CD63, and CD81 observed at the level of individual sEVs. Scale bar 100 nm. (**F**) Comparison of vesicle diameters measured using orthogonal methods, including TEM-derived sEVs, NTA-derived sEVs, dSTORM-derived sEVs, and TEM-derived ILVs (Red dashed line showing 200 nm). Statistical analysis was performed using the Kruskal–Wallis test followed by Dunn’s multiple comparisons test. sEV diameters measured by NTA and dSTORM were significantly larger than ILV diameters measured by TEM, whereas no significant difference was observed between TEM-derived sEV and ILV measurements. (* *p* < 0.005, ** *p* < 0.01, *** *p* < 0.001).

To examine whether the size conservation of ILVs is reflected in the size of sEVs, HEK293T cells were used to isolate sEVs by differential ultracentrifugation and filtration. The isolated sEVs were characterized by TEM, NTA, and dSTORM. Representative TEM micrographs are shown in Figure 3A, in which sEVs exhibit a characteristic bilayer, cup-shaped morphology. Diameters of sEVs measured from TEM micrographs were estimated across five biological replicates, with the majority of sEVs observed to be smaller than 200 nm (Figure 3B). The mean ± SD size distribution of sEVs measured by NTA is shown in Figure 3C. The distribution exhibited a unimodal profile, with the highest particle concentration observed within the sub-200 nm range. Most detected particles were distributed between approximately 50 and 200 nm in diameter, with a gradual decrease in particle concentration above 200 nm. Comparison of sEV size measurements obtained by TEM and NTA revealed that sEVs derived from the same cell source and biological replicates were significantly larger when measured by NTA than by TEM (Figure 3D). Phenotypic characterization of sEVs was performed using dSTORM. Representative images are shown in Figure 3E, in which individual sEVs were detected as single clusters. Phosphatidylserine affinity labeling (PAN-EV) was used to delineate the sEV membrane, and the presence of the canonical tetraspanins CD9, CD63, and CD81 was also detected by a combined tetraspanin antibody mix. Co-localization of PAN-EV signals with the tetraspanin trio was observed at the level of individual sEVs. Diameters obtained from automated EV acquisition using CODI were compared with diameters measured by TEM, NTA, and dSTORM. In addition, the size of the sEVs measured by TEM, NTA, and dSTORM showed no significant difference and no difference when compared with the mean ILV diameter from each study measured (Figure 3F).

## 3. Discussion

In this study, membrane-bound, roughly spherical vesicles located within the lumen of an MVB were operationally classified as ILV. While this was performed to systematically assess the size of ILVs, it is possible that a small fraction of these ILVs may be hard to distinguish from resembling structures found in other endolysosomal or autophagic compartments, such as lysosomes or amphisomes, and indeed, there is some evidence that these other compartments can give rise to EVs [14,69].

Despite characterization of ILVs and sEVs in individual reports, a unified quantitative framework defining ILV size across species, imaging modalities, and experimental contexts has not been established. Notably, the MISEV2018 guidelines do not specify a quantitative size range for ILV-derived exosomes or sEVs, whereas MISEV2023 indicates their size as generally smaller than 200 nm [70,71]. Importantly, this size claim lacks primary data or citations. Therefore, the present study provides empirical support for this proposed boundary. Although biophysical constraints governing ILV formation have been proposed [72] and ILV sizes have been reported in specific systems [20], these observations have not been systematically integrated across taxa or methodologies. In the present study, this gap was addressed through the integration of literature-based meta-analyses and direct comparison of ILV dimensions with sEV sizes measured using independent approaches. Thus, our study represents the first detailed evaluation of ILV size conservation across species and imaging platforms, and their comparison to sEVs.

Because the initial meta-analysis relied primarily on TEM-based measurements, known technical limitations associated with this modality were considered. TEM analysis can be influenced by fixation, dehydration, and staining artifacts, potentially biasing size estimation [73]. To further address this concern, a second meta-analysis focusing on cryo-EM studies was conducted. Cryo-EM has been widely recommended for EV imaging due to improved preservation of near-native morphology and reduced fixation-related artifacts [13,74,75]. Cryo-EM measurements likewise showed that ILV diameters were predominantly below 200 nm, with only one ILV of 205.5 nm observed. These findings suggest that the observed size distribution is unlikely to arise solely from methodological artifacts.

A notable limitation in previous ultrastructural studies concerns the discrimination between MVBs and amphisomes—hybrid organelles formed through fusion of MVBs with autophagosomes. Although both compartments contain intraluminal vesicles with comparable morphological appearance in electron micrographs, reliable distinction requires integration of molecular markers of the autophagic pathways [14,69]. Moreover, detailed ultrastructural characterization of amphisomes remains relatively limited, further constraining confident assignment of these compartments in conventional EM datasets.

The conserved upper size limit of approximately 200 nm of ILVs across species and imaging platforms is consistent with biophysical constraints governing ESCRT-mediated ILV biogenesis. Inward budding of the endosomal membrane requires high curvature and protein crowding, conditions that favor vesicle diameters in the range of approximately 30–150 nm [72]. ESCRT III-mediated neck constriction further enforces size uniformity, while geometric packing constraints within MVBs, typically measuring approximately 300–1000 nm in diameter, favor the formation of multiple smaller ILVs. Consistent with these principles, median ILV diameters of approximately 40–120 nm have been reported across taxa [76].

An additional source of heterogeneity may originate at the level of ILV biogenesis. Electron microscopy reveals that ILVs within a single MVB can differ markedly in diameter and electron density, suggesting the coexistence of multiple vesicle subpopulations prior to secretion. This intravesicular heterogeneity may contribute to the broad size distributions reported for sEVs in conditioned media by NTA and qNano and supports the notion that sEVs represent a structurally and functionally diverse population rather than a uniform entity. Notably, uniform ILV size distributions within an intracellular vesicle may raise consideration of potential virus particles [13]. The observed intravesicular ILV heterogeneity contributes to the broad size distributions reported for sEVs in conditioned media by NTA and qNano and supports the notion that sEVs may represent a structurally and functionally diverse population rather than a uniform entity.

To further examine the relationship between ILVs and their secreted counterparts, HEK293T cells were selected for downstream sEV analysis due to their high transfection efficiency, robust EV yield, and scalability [77,78]. Because sEVs released following MVB or amphisome limiting membrane plasma membrane fusion are considered secreted ILVs, comparison of ILV and sEV size distributions provides experimental support for their endosomal origin. sEVs isolated from HEK293T cells by differential ultracentrifugation were characterized using TEM, NTA, and dSTORM microscopy. Across all three orthogonal approaches, sEV diameters consistently remained below 200 nm, with closely concordant size distributions.

Taken together, the cumulative evidence supports that EVs larger than approximately 200 nm are unable to arise from ILVs and instead are derived from ectosomal shedding at the plasma membrane. This addresses a knowledge gap in EV heterogeneity, aiding standardization in MISEV guidelines and future biogenesis models. Thus, the present study provides a quantitative framework for ILV size conservation that aids in distinguishing endosome-derived sEVs from other EV subtypes in future studies.

## 4. Materials and Methods

### 4.1. Meta-Analysis of Intraluminal Vesicle Dimensions from Transmission Electron Microscopy

A meta-analysis was conducted to evaluate the size distribution of intraluminal vesicles (ILVs) identified in transmission electron microscopy (TEM) micrographs. A systematic literature search was performed in the PubMed database on 19 January 2026, using the following search strategy: (exosome AND TEM) OR (small + EV AND TEM) OR (MVB AND TEM) OR (ILV AND TEM). The initial search yielded 895 records. Studies were included in the analysis if they met the following criteria: (1) original research articles presenting multivesicular body (MVB) TEM micrographs with clearly visible intraluminal vesicles; (2) explicit measurements or quantifiable data of ILV dimensions; and (3) presence of scale bars or dimensional calibration references in micrographs. Studies lacking any of these criteria were excluded (*n* = 855). Following the application of inclusion and exclusion criteria, 40 original research articles were selected for meta-analysis (Figure 1A).

### 4.2. Meta-Analysis of Intraluminal Vesicle Dimensions from Cryo-Electron Microscopy

A second comprehensive meta-analysis was undertaken to assess ILV size distributions identified in cryo-EM datasets. A systematic literature search was conducted in the PubMed database on 20 December 2025, using the following search query: (cryo-EM OR cryo-ET) AND (“multivesicular body” OR “multivesicular bodies” OR intraluminal). The initial search retrieved 11 records. Studies were included if they satisfied the following criteria: (1) original research articles containing MVB cryo-EM or cryo-ET micrographs with resolved intraluminal vesicles; (2) quantifiable measurements of ILV dimensions; and (3) calibration references enabling dimensional analysis. Articles, which did not meet these criteria, were excluded (*n* = 3). Following screening, 8 original research articles met the inclusion criteria and were included in the meta-analysis (Figure 2A).

### 4.3. Image Analysis

All ILV and sEV diameter measurements derived from TEM, cryo-EM, and in situ experiments were quantified using ImageJ (version 1.54g). ILVs were selected based on the presence of roughly spherical, membrane-bounded structures observed within multivesicular bodies. For each micrograph, the image scale was first calibrated using the scale bar provided in the original figure. Individual vesicles were then manually outlined using the freehand selection tool, and each selected particle was added to the ROI Manager to ensure traceability and avoid duplicate measurements (Supplementary Information Figure S1). Vesicle size was quantified using the Feret diameter measurement for each ROI, and all measurements were exported for downstream analysis (Figure 1B).

### 4.4. Cell Culture

Human embryonic kidney (HEK293T) cells (Catalogue No. 12022001, ECACC, Salisbury, UK) were used in this study. Cells were cultured in Dulbecco’s Modified Eagle’s Medium (Catalogue No. D6046; Merck Group, Darmstadt, Germany) supplemented with 10% fetal bovine serum (Catalogue No. 10270106; Thermo Fisher Scientific, Waltham, MA, USA) and antibiotic–antimycotic solution (Catalogue No. A5955; Merck Group, Darmstadt, Germany). Cultures were maintained at 37 °C in a humidified atmosphere containing 5% CO_2_ (≥90% humidity). Cells were passaged three times per week. Polymerase chain reaction (PCR) was routinely performed to assess mycoplasma contamination using the following primers: GTTTGATCCTGGCTCAGGAYDAAC and GAAAGGAGGTRWTCCAYCCSCAC [79].

### 4.5. Isolation of Small Extracellular Vesicles from Cell Culture Supernatants

HEK293T cells were seeded at a density of 5 × 10^6^ cells per mL and incubated for 24 h in serum-free media (20 mL per 150 mm culture plate). sEVs were isolated using a differential centrifugation protocol with modifications to a previously described method [80]. The conditioned medium was subjected to sequential centrifugation and filtration steps to enrich for sEVs. Cells were first removed by centrifugation at 500× *g* for 10 min at room temperature. The resulting supernatant was passed through a 5 µm pore size filter (Merck Group, Darmstadt, Germany) and centrifuged at 2000× *g* for 20 min at 4 °C to remove very large extracellular vesicles. The supernatant was then filtered through a 0.8 µm pore size filter (Merck Group, Darmstadt, Germany) and centrifuged at 14,000× *g* for 40 min at 4 °C to remove large extracellular vesicles. The resulting supernatant was filtered through a 0.2 µm pore size filter (Merck Group, Darmstadt, Germany) and subjected to ultracentrifugation at 100,000× *g* for 2.5 h at 4 °C using an Optima XPN-100 ultracentrifuge (Beckman Coulter, Inc., Brea, CA, USA) to pellet sEVs. The resulting pellet was washed by resuspension in sterile phosphate-buffered saline (PBS) and subjected to an identical ultracentrifugation step. The final sEV pellet was resuspended in 1× PBS for subsequent analyses.

### 4.6. Transmission Electron Microscopy Imaging of Small Extracellular Vesicles

For TEM imaging of sEVs, we applied a previously described method [81]. Briefly, samples were fixed in 2% paraformaldehyde (paraformaldehyde, powder, 95%, 158127, Sigma Aldrich, St. Louis, MO, USA) in 1× PBS for 30 min at 4 °C. Then, they were incubated for 15 min on low, discharged at 7.2 V for 60 s, and carbon-coated on 100 mesh copper grids using a BalTec MED 020 Coating System (Baltec, Pfäffikon, Switzerland). After washing with 1× PBS and fixing with glutaraldehyde 1% (Sigma-Aldrich, St. Louis, MO, USA) for 5 min at 4 °C. Grids were washed again and dried with filter paper. The grids were treated with 2% aqueous uranyl acetate (Sigma-Aldrich, St. Louis, MO, USA) for 2 min to generate negative staining. Samples were washed and dried, then analyzed with FEI Tecnai G2 Spirit TEM (Thermo Fisher Scientific, Waltham, MA, USA) equipped with a Morada digital camera (Olympus Soft Image Solutions GmbH, Muenster, Germany). All TEM procedures were undertaken at the University of Valencia’s Electron Microscopy facility.

### 4.7. Nanoparticle Tracking Analysis

Size distribution and particle concentration of sEVs were determined using nanoparticle tracking analysis (NTA) on a ZetaView PMX120 instrument (Particle Metrix, Meerbusch, Germany). Samples were diluted in phosphate-buffered saline (PBS, 1×) to achieve optimal particle concentrations (50–200 particles per frame). Analysis was performed across 11 distinct positions on each sample, with two sequential measurement cycles acquired per position for data averaging and quality assurance. Instrument settings were optimized as follows: camera shutter speed, 100; camera sensitivity, 80; and frame rate, 30 frames per second. Data were processed and analyzed using the instrument’s integrated software, which automatically tracks individual particle trajectories to derive size distributions and concentration estimates.

### 4.8. Super Resolution Microscopy

Phenotyping of sEVs was performed using direct stochastic optical reconstruction microscopy (dSTORM) combined with the EV Profiler 2 assay platform (Oxford Nanoimaging, Oxford, UK). Super-resolution images of sEVs were acquired using a Nanoimager S microscope (Oxford Nanoimaging, Oxford, UK) equipped with a 100×, 1.4 numerical aperture oil immersion objective, an XYZ closed-loop piezo stage, and a single-molecule localization microscopy (SMLM) system featuring four laser lines and two spatially separated emission channels (split at 640 nm). Sample preparation followed the manufacturer’s protocol. The EV Profiler 2 assay chip surface was initially coated with surface reagent, followed by application of the Tetraspanin Trio capture reagent conjugated to biotinylated capture molecules. sEV samples (concentration range 10 × 10^9^–10 × 10^11^ particles per mL) were applied to the functionalized chip surface and incubated for 75 min at 30–45 rpm on a laboratory rocker for immobilization. Following immobilization and fixation, the surfaces were stained sequentially with Pan-EV Detection (488 nm) and Tetraspanin Trio Detection (561 nm) probes. Post-fixation was performed following staining, and the chip surface was incubated with dSTORM imaging buffer prior to image acquisition. Image acquisition was conducted in dSTORM mode using total internal reflection fluorescence (TIRF) microscopy for sequential dual-channel imaging. The 488 nm and 561 nm laser lines were operated at 84% and 45% power, respectively. Fluorescence localization data were processed and analyzed using the CODI platform (https://alto.codi.bio/, accessed on 7 December 2025) with AutoEV Profiler essentials acquisition settings. Localization clusters comprising ≥10 individual localizations were designated as sEVs. sEVs were classified as marker positive when ≥10 individual localizations were detected in a cluster in the corresponding detection channel.

### 4.9. Statistical Analysis

Descriptive statistics, including mean, standard deviation (SD), standard error of the mean (SEM), and lower and upper limits of the 95% confidence interval (CI), were calculated for all datasets. Frequency distributions were generated where appropriate, and Gaussian fitting was applied to visualize central tendencies without inferential interpretation. Statistical comparisons between multiple groups were performed using ordinary one-way analysis of variance (ANOVA) followed by Tukey’s multiple comparisons test. Pairwise comparisons between two groups were performed using unpaired two-tailed *t*-tests with Welch’s correction when variances were unequal. For non-parametric comparisons involving multiple groups, the Kruskal–Wallis test followed by Dunn’s multiple comparisons test was applied. Statistical significance was defined as *p* < 0.05, and significance levels are indicated in figures using asterisks (* *p* < 0.05, ** *p* < 0.01, *** *p* < 0.001, **** *p* < 0.0001). All statistical analyses were performed using GraphPad Prism (version 10.3.1).

## Figures and Tables

**Table 1:** Summary of intraluminal vesicle (ILV) diameter measurements obtained from TEM-based studies included in the meta-analysis. This table summarizes quantitative ILV size measurements extracted from TEM micrographs across all studies included in the TEM-based meta-analysis. For each study, the organism examined, the number of ILVs measured (N), mean ILV diameter, standard deviation (SD), and lower and upper limits of the 95% confidence interval (CI) are reported. The graphical representation can be found in the Supplementary Information Figure S2A.

Sl No.	Studies	Species	*N*	Mean	SD	95% CI	Reference
1	Reibman J et al., 2002	human	13	89.9	21.99	76.61–103.2	[21]
2	Bache K et al., 2003	human	9	64.02	17.58	50.51–77.54	[22]
3	Luhtala N et al., 2004	human	7	128.4	4.173	124.5–132.2	[23]
4	Chung Tse Y et al., 2004	plant	11	74.68	19	61.92–87.44	[24]
5	Wang J et al., 2007	plant	24	139.9	17.56	132.5–147.3	[25]
6	Darehshouri A et al., 2008	green alga	14	106.4	24.94	92.01–120.8	[26]
7	Altick A et al., 2009	rat	17	72.2	12.8	65.62–78.78	[27]
8	Sahoo S et al., 2011	human	6	49.17	12.27	36.29–62.04	[28]
9	Eden E et al., 2012	human	22	131.4	5.055	129.2–133.7	[29]
10	Zeigerer A et al., 2012	mouse	6	59.75	10.66	48.56–70.94	[30]
11	Chivet M et al., 2012	rat	24	59.59	12.81	54.18–65	[31]
12	Wang J et al., 2012	plant	12	142.6	9.802	136.4–148.8	[32]
13	Wanner G et al., 2013	green alga	27	152.6	16.27	146.2–159	[33]
14	Edgar J et al., 2013	human	39	57.99	16.34	52.7–63.29	[34]
15	Adamakis IS et al., 2013	Plant	25	160.8	19.7	152.6–168.9	[35]
16	Hofmann D et al., 2014	human	10	87.35	20.81	72.46–102.2	[36]
17	Nagashima S et al., 2014	human	30	51.61	20.07	44.12–59.11	[37]
18	Soonsawad P et al., 2014	human	5	95.32	46.92	37.07–153.6	[38]
19	Santo N et al., 2014	planktonic crustacean	7	163	15.38	148.8–177.2	[39]
20	Petrizzo A et al., 2015	human	44	74.24	37.23	62.92–85.56	[40]
21	Mazzeo C et al., 2015	human	7	94.9	28.1	68.91–120.9	[41]
22	Hyenne V et al., 2015	Caenorhabditis elegans	59	61.05	10.34	58.36–63.75	[42]
23	Chihanga T et al., 2018	human	64	66.37	25.16	60.09–72.66	[43]
24	Chen Q et al., 2018	mouse	8	144.1	13.92	132.5–155.8	[44]
25	Lammel T et al., 2019	Fish	44	167.5	18.5	161.9–173.2	[45]
26	Bai X et al., 2019	Fish	113	97.34	47.23	88.54–106.1	[46]
27	Zhao DY et al., 2019	human	21	93.97	15.49	86.92–101	[47]
28	Adamakis ID S et al., 2019	plant	27	125.7	26.02	115.4–136	[48]
29	Rupp U et al., 2019	Isopods	17	41.62	6.994	38.02–45.21	[49]
30	Vistro WA et al., 2019	turtle	23	133.3	30.85	119.9–146.6	[50]
31	Zhang XW et al., 2020	human	17	161.9	19.61	151.9–172	[51]
32	Belli M et al., 2020	mouse	4	159.3	20.07	127.3–191.2	[52]
33	Campos C et al., 2020	fungus	10	142.4	26.49	123.5–161.3	[53]
34	Qu W et al., 2020	goat	9	119.6	34.57	93.04–146.2	[54]
35	Polat I et al., 2020	insect	8	141.9	32.28	114.9–168.9	[55]
36	Steiner P et al.,2021	green alga	7	103.7	15.56	89.31–118.1	[56]
37	Santo N et al., 2021	plant	7	103	35.49	70.21–135.9	[57]
38	Chatzimeletiou K et al., 2022	human	24	110.8	14.76	104.6–117	[58]
39	Zhu Z et al., 2024	Fish	36	110.1	25	101.7–118.6	[59]
40	Bergner T et al., 2024	Human	12	122	27.77	104.4–139.7	[60]
	Average		21.725	106.54	20.89		

**Table 2:** Summary of intraluminal vesicle (ILV) diameter measurements obtained from cryo-EM-based studies included in the meta-analysis. This table summarizes quantitative ILV size measurements extracted from cryo-EM micrographs across all studies included in the cryo-EM-based meta-analysis. For each study, the organism examined, number of ILVs measured (N), mean ILV diameter, standard deviation (SD), and lower and upper limits of the 95% confidence interval (CI) are reported. The graphical representation can be found in the Supplementary Information Figure S2B.

Sl No.	Studies	Organism	*N*	Mean	SD	95% CI	Reference
1	Ma D et al., 2022	Mouse	13	58.4	12.33	50.94–65.85	[61]
2	Tavakol A et al., 2025	Mammalian cells	5	60.3	9.262	48.8–71.8	[62]
3	Flores M D et al., 2025	Human	5	46.24	8.647	35.5–56.97	[63]
4	Tau C L et al., 2025	Rat	17	46.38	8.675	41.92–50.85	[64]
5	Sykora U M et al., 2025	Human	5	85.62	24.07	55.74–115.5	[65]
6	Groe J et al., 2025	Monkey	9	153.3	34.58	126.7–179.9	[66]
7	Alcantara C L et al., 2025	Fungi	20	43.68	5.074	41.31–46.06	[67]
8	Ning J et al., 2025	Mouse	12	69.03	12.76	60.93–77.14	[68]
	Average		10.75	70.4	14.4		

**Table 3:** Comparison of intraluminal vesicle (ILV) diameter measurements obtained from literature-based meta-analyses and in situ experiments. This table summarizes ILV diameter measurements derived from TEM-based literature meta-analysis, cryo-EM-based literature meta-analysis, and TEM-based in situ validation using cell lines. For each dataset, the average number of ILV measured for each study (N), the number of studies or experimental conditions analyzed (n), mean ILV diameter, standard deviation (SD), and lower and upper limits of the 95% confidence interval (CI) are reported.

	*n*	*N*	Mean	SD	95% CI
Literature (ILV by TEM)	40	21.725	106.54	20.89	85.68–113
Literature (ILV by Cryo-EM)	8	10.75	70.40	14.40	40.02–100.7
Average of 48 studies	48	19.90	100.50	39.12	89.15–111.9

## Data Availability

The original contributions presented in this study are included in the article/Supplementary Materials. Further inquiries can be directed to the corresponding author.

## Supplementary Materials

The following supporting information can be downloaded at: https://www.mdpi.com/article/10.3390/ijms27073176/s1.
