# Supplementary material for: Exosome Biogenesis: Meta-Analysis of Intraluminal Vesicle Size Across Species
Source: Int J Mol Sci. 2026 Mar 31;27(7):3176. doi: 10.3390/ijms27073176 (PMC13073769; doi:10.3390/ijms27073176)
Supplement: Supplementary file 1 [file ijms-27-03176-s001.zip › ijms-4141942-supplementary.pdf]

Additional information for

Exosome biogenesis: meta-analysis of intraluminal vesicle size across species

Sayam Ghosal et al.

*Corresponding author email: xabieruf@gmail.com

This PDF file includes:

Figures S1 to S2

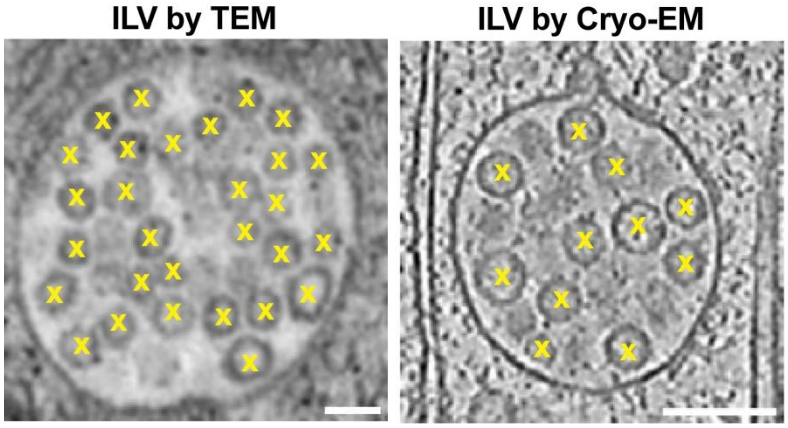

Figure S1. Determination of intraluminal vesicle (ILV).

Single representation of determination of ILV from TEM, cryo-EM. The TEM and Cryo-EM micrographs were adapted from Zhu et al., Fish and Shellfish Immunology (2024) (left) and Groen et al., Communications Biology (2025) (right). Scale bar 100 nm. The highlighted "X" in yellow represents single ILV accounted into these studies. For their diameter measurement the pixel-to-nanometer scale for each micrograph was calibrated using the original scale bar. ILVs were traced manually with the freehand selection tool, and each region of interest (ROI) was logged in the ROI Manager to maintain traceability and avoid repeated measurements. ILV size was calculated as the Feret diameter for every ROI, and the resulting values were exported for downstream analysis. Fig 1B provides a schematic overview of this measurement workflow.

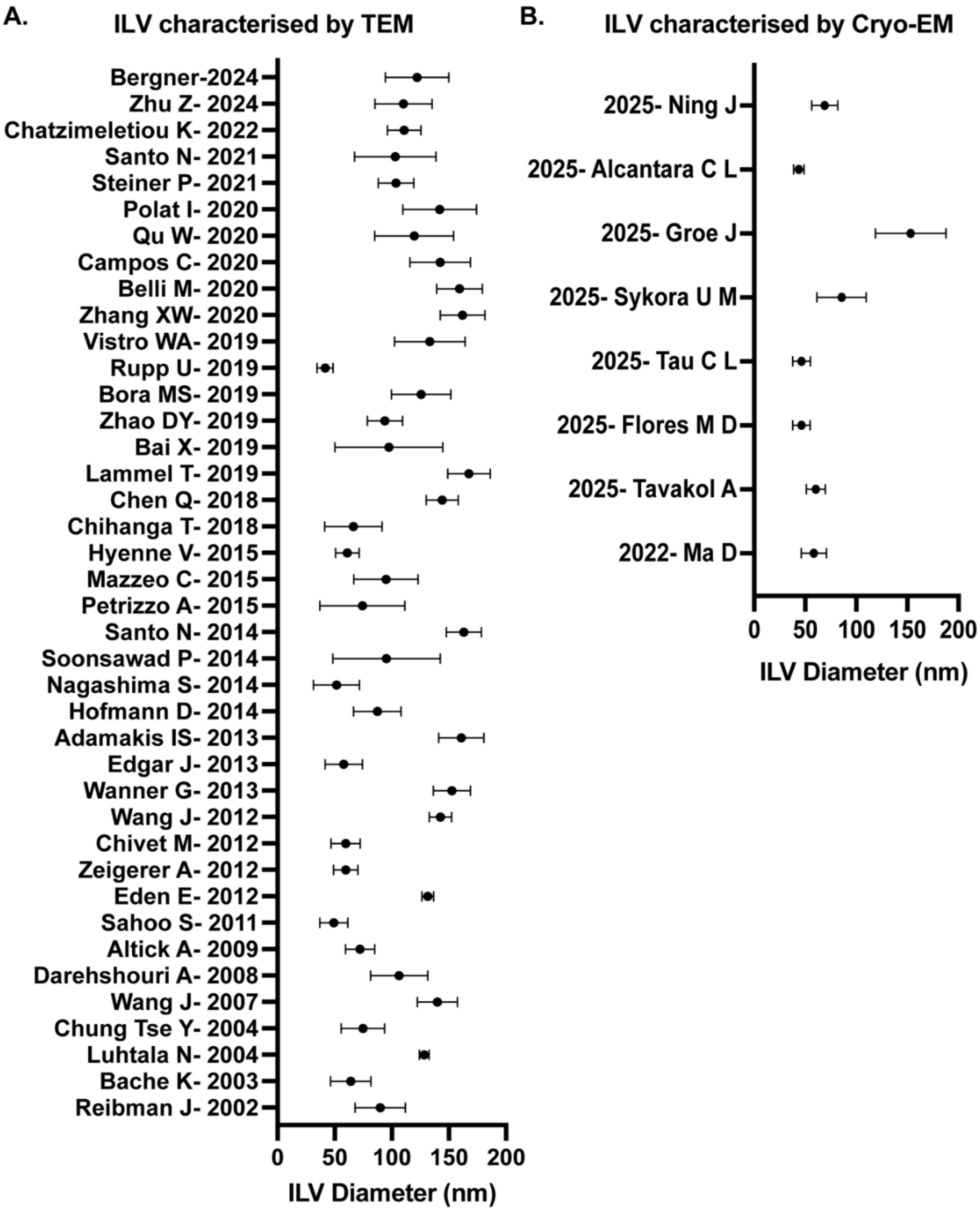

Figure S2. Reported intraluminal vesicle diameters measured by TEM and cryo-EM across published studies.

Reported intraluminal vesicle (ILV) diameters in multivesicular bodies measured by transmission electron microscopy (TEM) and cryo-electron microscopy (cryo-EM). (A) ILV diameters compiled from published TEM studies, plotted as mean ± standard deviation of the ILV measured per studies. (B) ILV diameters compiled from cryo-EM studies, plotted in the same manner to enable comparison with TEM-derived values.
